# Formal synthesis of non-fragile state-feedback digital controllers considering performance requirements for step response

**DOI:** 10.1038/s41598-022-19284-4

**Published:** 2022-09-14

**Authors:** Thiago Cavalcante, Iury Bessa, Eddie B. de Lima Filho, Lucas C. Cordeiro

**Affiliations:** 1 Graduate Program in Electrical Engineering, Federal University of Amazonas, Manaus, AM 69067-005 Brazil; 2 Department of Electricity, Federal University of Amazonas, Manaus, AM 69067-005 Brazil; 3 TPV Technology, Manaus, AM 69058-581 Brazil; 4 Department of Computer Science, The University of Manchester, Manchester, M13 9PL UK

**Keywords:** Electrical and electronic engineering, Computer science

## Abstract

This work describes an approach for synthesizing state-feedback controllers for discrete-time systems, taking into account performance aspects. The proposed methodology is based on counterexample-guided inductive synthesis (CEGIS), producing safe controllers based on step response performance requirements, such as settling time and maximum-overshoot. Controller candidates are generated through constrained optimization based on genetic algorithms. Each iteration that does not satisfy the initial system requirements is learned as a failed result and then used in another attempt. During the verification phase, it is considered the controller fragility to ensure deployable implementations. Such an approach assists the discrete-time control system design since weaknesses occur during implementation on digital platforms, where systems that meet design requirements are employed. The proposed method is implemented in DSVerifier, a tool that uses bounded (and unbounded) model checking based on satisfiability modulo theories. Experimental results showed that our approach is practical and sound regarding the synthesis of discrete state-feedback control systems that present performance requirements. It considers finite word-length effects, unlike other methods that routinely ignore them.

## Introduction

The design of digital controllers is one of the essential tasks regarding digital control systems^1^. In particular, such structures consist of sensors, controlled system, control algorithms, and actuators, which together seek to maintain the desired behavior of plants’ (controlled system) variables under control, i.e., they ensure the desired transient and steady-state responses^2^. The digital control theory aims to preserve some properties, based on discrete-time models, e.g., stability and robustness. Those properties are necessary for the correct operation of real plants through a digital controller; however, controlling continuous systems with digital controllers raises new challenges such as: round-off and quantization of samples and coefficients in digital controllers, due to finite word-length (FWL) implementations, can lead to overflow, poles and zeros sensitivity, limit-cycle oscillation (LCO), approximation errors, and other types of deviations from expected behavior, which might cause system instability and performance degradation^3,4^. Indeed, the sensitivity concerning implementation issues is called fragility^3^ and the design of control systems should address that issue, which drives the attention of control systems and verification communities.

Some initiatives applying formal verification to dynamic control systems have been developed^4–8^ in the past years which includes verification methods that take into account implementation aspects and determine uncertain linear-system stability regarding digital controllers^4^, schemes that address validation of robustness at both model and code level^5^, approaches for verifying digital filters w.r.t overflow and approximation errors, due to quantization and round-off effects^6^, and controller synthesis, in an attempt to generate correct-by-construction elements^8^. Moreover, there exists also an approach that aims to formally verify whether a digital control system meets performance requirements, such as settling time and maximum overshoot, using both open- and closed-loop forms and considering FWL effects^9^. Such a work gathered on a unified framework, fragility aspects, and performance requirements, making it possible to check systems and ensure that they could be promptly deployed on real scenarios.

In recent years, the control-system literature brought some studies that tackle program synthesis, which is a technique used for finding entities that satisfy user intent expressed in some form of constraints^10^. Besides, studies that aim to formally synthesize controllers can also be found^11,12^. Among other options, a program synthesis technique called counterexample guided inductive synthesis (CEGIS) is of particular interest, given its main features. It is an approach that determines unknown parameters inside partial programs so that the resulting elements satisfy correctness properties^13^. Some studies have been done using CEGIS for performing synthesis^14^ and such a work can be successfully used for designing stable controllers for continuous plants; however, when FWL effects are considered, a further verification step should then be performed, given the possibility of overflow, LCO, and instability, for instance^15^. Indeed, it suggests that an additional verification phase could be integrated into the mentioned CEGIS approach and then used to generate elements already suitable for a given FWL scenario. Work that applies CEGIS for stabilizing controllers can also be found in literature^13^, and, as a consequence, one may argue that CEGIS could also be combined with performance-requirement verification, such as overshoot and settling time, to satisfy a specific behavior.

The idea of combining fragility verification, performance requirement, and CEGIS served as inspiration for the present work, which introduces a formal methodology for synthesizing a controller while evaluating performance requirements in digital control systems by using the CEGIS technique and genetic algorithms (GAs)^16^ to generate controller candidates. The CEGIS technique has two main stages, a verification step and a synthesizing one (learning step), and both are tackled here. The first step is implemented as part of another recently published work^9^ and it was enhanced here by integrating a formal verification methodology into an automatic controller synthesizer as a part of our CEGIS methodology designed in the presented work. Regarding the second step, we have devised an optimization problem and then solved it using GAs to generate candidates for the associated CEGIS engine. The resulting scheme was implemented in a bounded (and unbounded) model checker named as DSVerifier^17^, which is a tool able to find FWL problems in digital controllers and filters. The reasons behind that are multifold, which includes using a validating framework, a practical tool for designing digital controllers, and the availability of base modules already suitable for formal checking.

Additionally, according to a recent survey on the state-of-the-art of verification and synthesis methods for cyber-physical systems^18^, most papers published in the area in the past ten years only study the verification of performance properties over mathematical representations of digital controllers. However, a top-to-bottom synthesis process of digital controllers will need to cover various aspects, including, for example, the hardware platform with which the digital controllers are implemented. Thus, there is a considerable gap between low- and high-level models and between engineering and theoretical research efforts. In summary, this paper makes the following original contributions in this research direction.

- We describe a novel approach for synthesizing open- and closed-loop linear time-invariant systems while considering performance requirements, system fragility, and FWL effects in fixed-point representations of controllers. As a result, this paper is the first to address the problem of designing correct-by-construction controllers, which takes into account performance requirements, encoded in an automated CEGIS-based methodology, which can handle different FWL formats.
- Experimental results show that our approach can effectively synthesize settling time and overshoot specifications in control applications suffering from FWL effects. Our results also show the provision of a system model description capable of being integrated into the setup of a GA-based optimization, leading to substantial improvements for synthesizing digital controllers for different FWL formats.

The remainder of this paper is organized as follows. In “Preliminaries” section presents basic concepts of digital dynamic systems, FWL effects in digital systems, and CEGIS, while “Verifying non-fragile performance specification requirements” section shows the verification methodology employed for evaluating digital control systems. In “Non-fragile synthesis for performance requirements” section describes the proposed method for synthesizing digital controllers, considering FWL effects. In “Experimental evaluation” section, in turn, reports experiments regarding a set of benchmarks and discusses their results. Next, “Related work” section presents a summary of studies related to formal verification and synthesis applied to system controllers. Finally, “Conclusion” section draws our conclusions.

## Preliminaries

A system can be seen as a process with inputs and outputs, which is usually simplified to only one input and one output. In particular, a system produces a transformation regarding an input signal *x*(*t*), which then generates an output one *y*(*t*) that is usually described by a mathematical operator $S\{\cdot \}$. Controllers are employed for modifying a system to make it present a given desirable behavior. This section aims to introduce fundamental concepts of digital systems, design problems, and tools necessary for developing a methodology based on CEGIS that can produce correct-by-construction controllers.

### Digital dynamic systems

The state-space representation of a single-input single-output (SISO) linear time-invariant (LTI) system $\Omega$ controlled by a state feedback controller is described as

1 $$\begin{aligned} \Omega :{\left\{ \begin{array}{ll} x(k+1)=Ax(k)+Bu(k),\\ y(k)=Cx(k)+Du(k),\\ u(k)=r(k)-Kx(k), \end{array}\right. } \end{aligned}$$

where $A\in {\mathbb {R}}^{n\times n}$, $B\in {\mathbb {R}}^{n\times 1}$, $C\in {\mathbb {R}}^{1\times n}$, and $D\in {\mathbb {R}}$ are the state-space realization matrices (*A*, *B*, *C*, *D*) of $\Omega$, $K\in {\mathbb {R}}^{1\times n}$ is a state-feedback controller, $u(k)\in {\mathbb {R}}$ is the control signal, $y(k)\in {\mathbb {R}}$ is the system’s output signal, $r(k)\in {\mathbb {R}}$ is a control reference signal, and $x(k)\in {\mathbb {R}}^{n}$ is a state vector.

**Figure 1:**
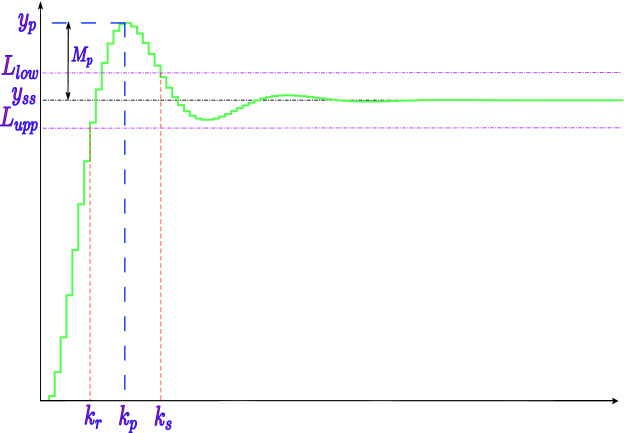
We illustrate the generic step response of a digital control system represented by Eq. (1), which emphasizes the settling time region bounded by $L_{\mathrm {upp}}$ and $L_{\mathrm {low}}$ and the maximum overshoot $M_{\mathrm {p}}$ occurring at $k_{\mathrm {p}}$.

Figure 1 illustrates the generic step response of (1), where one may notice the area bounded by $L_{\mathrm {upp}}$ and $L_{\mathrm {low}}$, i.e., the settling time region $\Pi$, where a signal remains within $p\%$ of its final value $y_{\mathrm {ss}}$, which happens for the first time at $k_{\mathrm {r}}$ (reach time) and definitively begins at $k_{\mathrm {s}}$ (settling time). Moreover, there exists a maximum overshoot $M_{\mathrm {p}}$ occurring at $k_{\mathrm {p}}$.

### FWL effects in digital systems

Usually, control systems are designed with real valued coefficients. Nonetheless, system parameters and signal variables are implemented with limited word-length through hardware registers^19^, which gives rise to FWL effects due to truncation and round-off errors^3,20^.

**Figure 2:**
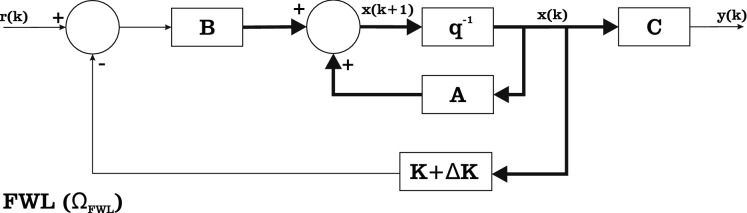
We illustrate the block diagram of a discrete-time feedback control system with FWL effects, where $\Delta K$ denotes FWL effects.

Indeed, controllers of a digital control system are heavily impaired by FWL effects^9^, as one may notice in Fig. 2, which illustrates the block diagram of a discrete-time feedback control system, where $\Delta K$ denotes FWL effects. Consequently, such variations can influence a system’s response and its required settling time $t_{\mathrm {sr}}$, which could be neglected due to underestimation of FWL effects^21,22^. As indicated by Cavalcante et al.^9^, FWL effects can be formulated as

2 $$\begin{aligned} {\mathscr{F}}{\mathscr{W}}{\mathscr{L}}_{\langle I,F \rangle }[\cdot ]:{\mathbb {R}}^{m\times n} \rightarrow {\mathbb {R}}^{m\times n}_{Q}~, \end{aligned}$$

where ${\mathbb {R}}^{m\times n}_{Q}$ is the discrete set of matrices $m\times n$ composed by elements of ${\mathbb {R}}^{m\times n}$, which can be represented in the fixed-point format $\langle I,F \rangle$, while *I* and *F* are the number of bits of integer and fractional parts, respectively. As the coefficients of *K* are subject to FWL effects^9^, a state-space representation can be written as

3 $$\begin{aligned} \Omega _{\mathrm {FWL}}:{\left\{ \begin{array}{ll} x(k+1)=Ax(k)+Bu(k)\\ y(k)=Cx(k)+Du(k)\\ u(k)=r(k)-K_{\mathrm {FWL}}x(k) \end{array}\right. }, \end{aligned}$$

where the matrix $K_{\mathrm {FWL}}:={\mathscr{F}}{\mathscr{W}}{\mathscr{L}}_{\mathrm {\langle I;F \rangle }}[K]$ is the controller gain considering FWL effects.

The FWL effects due to round-offs and quantization have been investigated in the recent literature^4,15,23^ which indicates that they might lead to imprecision and even instability. Most of the non-fragile control techniques describes FWL effects in the digital controller implementation as parametric uncertainties and adopt robust control techniques to ensure the closed-loop properties^20^. However, the FWL effects might be accurately computed if the implementation characteristics (e.g., number of fractional and integer bits of fixed-point representation) are known. This paper adopts the accurate FWL effects on fixed-point implementations of state-feedback controllers as described in^23^.

Additionally, other control issues related to FWL effects are investigated in the literature, e.g., overflows and limit cycle oscillations (LCOs)^15,24^. However, to the best of our knowledge, it is the first work to propose CEGIS-based design of state-feedback controllers considering the step response performance requirements.

### CounterExample-guided inductive synthesis

CEGIS is an iterative process for program synthesis that is becoming popular, where each iteration performs inductive generalization based on counterexamples provided by a verification engine. Typically, inductive generalization uses information about a limited number of inputs to make claims about all possible inputs in the form of candidate solutions^14^.

**Figure 3:**
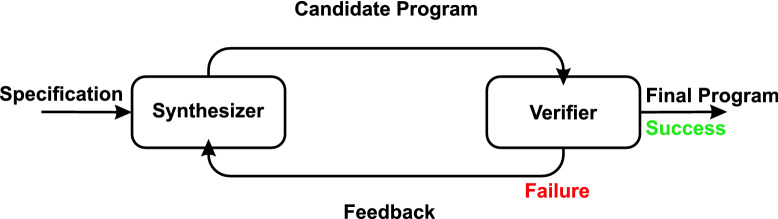
We illustrate the traditional CEGIS’ block diagram^9^, where the synthesizer provides candidate solutions, while the verifier checks whether these solutions meet the specification. We implement a learning algorithm in the synthesizer, responsible for refining candidate solutions based on feedback (counterexamples) provided by the verifier.

Figure 3 shows the typical CEGIS’ framework^14^ and consists of two main stages: synthesis and verification. The former performs the generation of a candidate program, which can presumably satisfy a given property. The latter, in turn, provides a way of checking requirement satisfiability regarding the same property, i.e., if it fails or succeeds.

In the synthesis stage, a learning algorithm exists responsible for refining candidate solutions based on counterexamples provided by a verification engine. The learning algorithm proceeds by searching the space of candidate concepts for one consistent with the examples seen so far. There may be several consistent concepts, but the employed search strategy determines the chosen candidate, then presented to a verification engine and checked against a correctness specification. If the current candidate is correct, the synthesizer terminates and outputs it; otherwise, the verification engine generates a counterexample, which informs the reason for this failure. Next, the same counterexample is forwarded to the learning algorithm, which adds it to a specific group and then repeats its search. It is possible that, after a given number of iterations, the learning algorithm is unable to find a consistent candidate concept, in which case the learning step and hence the overall CEGIS procedure fail^25^.

Although the CEGIS’ framework is well-defined, and all associated steps are clear and self-contained, there still exists room for improvement. For instance, in the synthesis stage, it is possible to generate candidates that present more chance to be correct, i.e., it is likely that they meet the input requirements, which may range from an auxiliary model to specific generation algorithms. In this work, we decided to formulate an optimization problem and solve it using GAs to generate candidates that meet design requirements in the form of constraints for an optimization problem. GAs perform a global search and then explore the search space using different kinds of crossover. Besides, as a controller generated by a GA already satisfies the constraints of the optimization problem (here, constraints are the same as requirements), the synthesis mechanism only needs to ensure that it behaves this way when operating under FWL effects.

The compliance with respect to the performance requirements by considering the FWL is checked in the verification step. It is possible to use formal verification approaches to check candidates, given that they are robust solutions and proved to be practical and effective^4,26^. In this paper, the verifier stage is performed based on the digital control system verification methodology presented in^9^, which is summarized in the next “Verifying non-fragile performance specification requirements” section. The synthesizer step proposes a GA-based optimization where the constraints are updated when a verifier stage indicates that the solution is invalid. A candidate controller generated by the GA is considered valid only if its quantized version $K_{\mathrm {FWL}}$ succeeds in the verification step. The former step is discussed in “Non-fragile synthesis for performance requirements” section.

## Verifying non-fragile performance specification requirements

In this study, we use a CEGIS synthesis technique to generate a controller to satisfy performance requirements. In this CEGIS implementation, we have two main stages: *synthesis* and *verification*, the latter being tackled in this section. Specifically, we show the synthesis schemes developed for settling time and maximum overshoot in the context of digital control systems.

### Maximum overshoot estimation

A formal parameter is needed to verify the maximum overshoot in a control system using formal methods, which involves property satisfiability. Regarding this, Algorithm 1 was developed, which describes a procedure to estimate $y_{\mathrm {p}}$, i.e., the maximum peak value in the response of a system, and $k_{\mathrm {p}}$, i.e., the sample where $y_{\mathrm {p}}$ is located, in order to find a formal approach to verify the maximum overshoot used in the proposed CEGIS approach.

Algorithm 1 was developed in a previous work^9^, thus it will be shortly discussed here. The main parameters of Algorithm 1 are defined as follows:

- $\nabla _{k}$ is the variation in the output signal;
- $y_{\nabla _{\mathrm {i}}}$ is the amplitude of the sample where the last gradient was detected;
- *i* is the sample index where this amplitude is located;
- $\xi$ is the number of detected peaks that are not of interest for the current analysis, i.e., false peaks smaller than the current one;
- *k* is the current sample number iteration.

As inputs for Algorithm 1, we have state matrices ${\mathbf {A}}$, ${\mathbf {B}}$, ${\mathbf {C}}$ and ${\mathbf {D}}$, controller ${\mathbf {K}}$ (if the analysis regards a closed-loop system), and system input *u*. Indeed, input data are used for intermediate calculations, such as obtaining *y*(*k*) and $y_\mathrm {ss}$, with their respective formulae. The outputs of Algorithm 1 are the maximum peak ($y_\mathrm {p}$) and the peak time ($k_{\mathrm {p}}$).
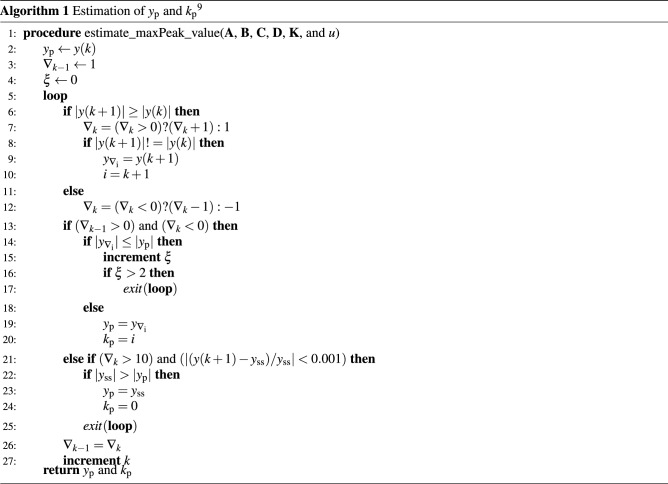


Finally, the maximum overshoot $M_{p}$ is computed as follows:

4 $$\begin{aligned} M_{p}=y_{p}-y_\mathrm {ss}. \end{aligned}$$

### Settling time invariant estimation

Cavalcante et al.^9^ provide a methodology to compute an invariant for settling time of discrete-time systems. In the verification literature, an invariant is a logical proposition which over-approximates the set of reachable states by allowing the reduction of the verification space^27^. In this paper, we find an upper-bound for the settling-time to allow pruning the verification space up to that bound. In summary, the following function based on the eigenvalues is defined:

5 $$\begin{aligned} {\overline{y}}(k)=y_{\mathrm {ss}}+{\overline{c}}{\overline{\lambda }}^{k}~, \end{aligned}$$

where ${\overline{c}}$ is a constant that makes ${\overline{y}}(k)$ enter the settling time region, based on the slowest eigenvalue ${\bar{\lambda }}$. Given the definition of the settling time itself and the heuristic function, the instant (${{\hat{k}}}$) a system enters its settling time region can be found with

6 $$\begin{aligned} \begin{aligned} {{\hat{k}}}={\lceil } \log _{{\overline{\lambda }}}\left( \frac{p}{100{\overline{c}}}y_{\mathrm {ss}}\right) {\rceil }, \end{aligned} \end{aligned}$$

where *p* indicates the setting time region, in percentage (please, see “Digital dynamic systems” section). A design procedure, with Eq. (5), $k_{\mathrm {p}}$, and $y_p$, could then obtain ${\overline{c}}$, which allows modeling of the settling time region dynamics, based on the slowest eigenvalue:

7 $$\begin{aligned} \begin{aligned} {\bar{c}}=&~\frac{y_{\mathrm {p}}-y_{\mathrm {ss}}}{{{\bar{\lambda }}}^{k_{\mathrm {p}}}}. \end{aligned} \end{aligned}$$

In a nutshell, the reasoning behind this mathematical formulation is that if the slowest eigenvalue indicates that the desired settling time is reached, i.e., ${\hat{k}} \le k_{\mathrm {s}}$, it is assured that the entire system meets this requirement, given that the other eigenvalues are faster. Nonetheless, when that does not happen, it is not assured that a given system does not meet $k_{\mathrm {s}}$, given the net result of the interaction among eigenvalues, which may then lead to a direct verification regarding output samples. Finally, the most direct use of ${\hat{k}}$ is as an invariant during verification procedures.

### Settling time and overshoot verification algorithms

Based on the estimates of the settling time-invariant and the maximum overshoot, verification algorithms for overshoot and settling time requirements are presented in^9^.

The overshoot verification technique^9^consists in checking if the actual percentage overshoot *PO* (as computed) is lower than or equal to the required percentage overshoot ($PO_{\mathrm {r}}$). If the latter is true, it consequently leads to a Verification SUCCESSFUL; otherwise, Verification FAILED is reported. In summary, $y_{\mathrm {ss}}$ and $PO_{\mathrm {r}}$ are the necessary inputs for this algorithm, which performs a simple comparison.

In order to find the overshoot percentage, based on the maximum overshoot ($M_{\mathrm {p}}$) shown in Fig. 1, one can use^9^

8 $$\begin{aligned} \begin{aligned} PO =&~ 100\times \frac{M_{\mathrm {p}}}{y_{\mathrm {ss}}}, \end{aligned} \end{aligned}$$

which can be run after Algorithm 1.

The overshoot verification consists basically in checking if the computed percentage overshoot *P*.*O*. (Eq. 8) is lower than the required percentage overshoot ($P.O._{\mathrm {r}}$), which, if true, consequently leads to a Verification SUCCESSFUL.

Regarding the settling time verification algorithm, we assume that the main algorithm has access to all parameters used by it and produces Verification SUCCESSFUL or Verification FAILED, as output. There also exists a straightforward companion algorithm (not shown here) that receives, as inputs, system specifications (state-space matrices, input samples, and a controller) and produces $k_\mathrm {r}$ (instant where that output reaches the settling time region), as output. This algorithm is useful when $y_p$ is in the settling time region (see the last conditional block of Algorithm 2).

The proposed procedure for verifying settling time, which was developed by Cavalcante et al.^9^, is described in Algorithm 2 and consists in first checking if ${\hat{k}}$, as computed with Eq. (6) (see “Settling time invariant estimation” section), is lower than the required settling time $k_{\mathrm {sr}}$, which promptly assures success. Nonetheless, if that is not the case, a system’s output must then be directly checked. Indeed, the invariant approach’s main advantage is based on the adopted heuristic function: no computation based on a system’s model is needed, which provides high speed and simplicity.
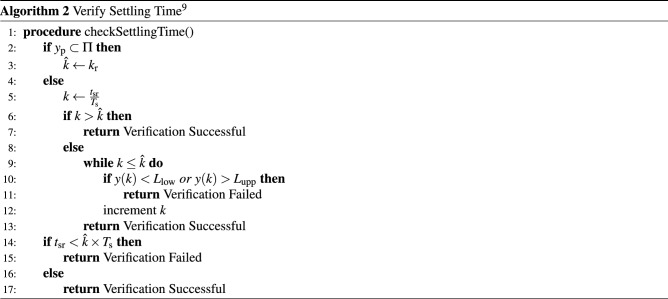


Notice that both Algorithms 1 and 2 can be used to verify systems with or without FWL effects. In order to guarantee the safety of the implemented controller, it is necessary to check the system $\Omega _{\mathrm {FWL}}$ with the quantized controller $K_{\mathrm {FWL}}$.

For a deeper understanding of the presented verification methodologies, we refer the reader to the work developed by Cavalcante et al.^9^.

## Non-fragile synthesis for performance requirements

In this section, we describe our proposed synthesis methodology based on the specification of performance requirements for digital control systems. The synthesis methodology is based on the CEGIS technique, with two stages: verification and learning (where a specific synthesizer is located), as illustrated in Fig. 3. In the learning stage, we generate a controller *K* to satisfy our performance requirements (settling time and/or overshoot), using GAs^16^, where we take system definitions and specifications as constraints. After generating *K*, a verification stage employs the verification approaches for settling time and overshoot on the system $\Omega _{\mathrm {FWL}}$ described in (3) and considering the FWL effects, as explained in “Settling time and overshoot verification algorithms” section. Then, it receives the mentioned controller *K* and verifies if the latter meets the initial requirements. If the presented procedure fails in that stage, it goes back to the learning step and keeps repeating that loop until it is successful regarding the associated verification.

In our CEGIS methodology, GAs perform a global search and explore search spaces using different kinds of crossover, i.e., combination procedures, afterwards^28^. That feature provides a great advantage to GAs regarding finding a solution, which is not necessarily the global optimal, for a given optimization problem and is very interesting regarding digital controllers due to the need for individual coefficient tuning. Moreover, as a controller generated by GAs already satisfies the constraints of the underlying optimization problem, in the CEGIS-based scheme proposed here, a synthesis mechanism only needs to ensure that it still meets the input requirements when operating under FWL effects.

**Figure 4:**
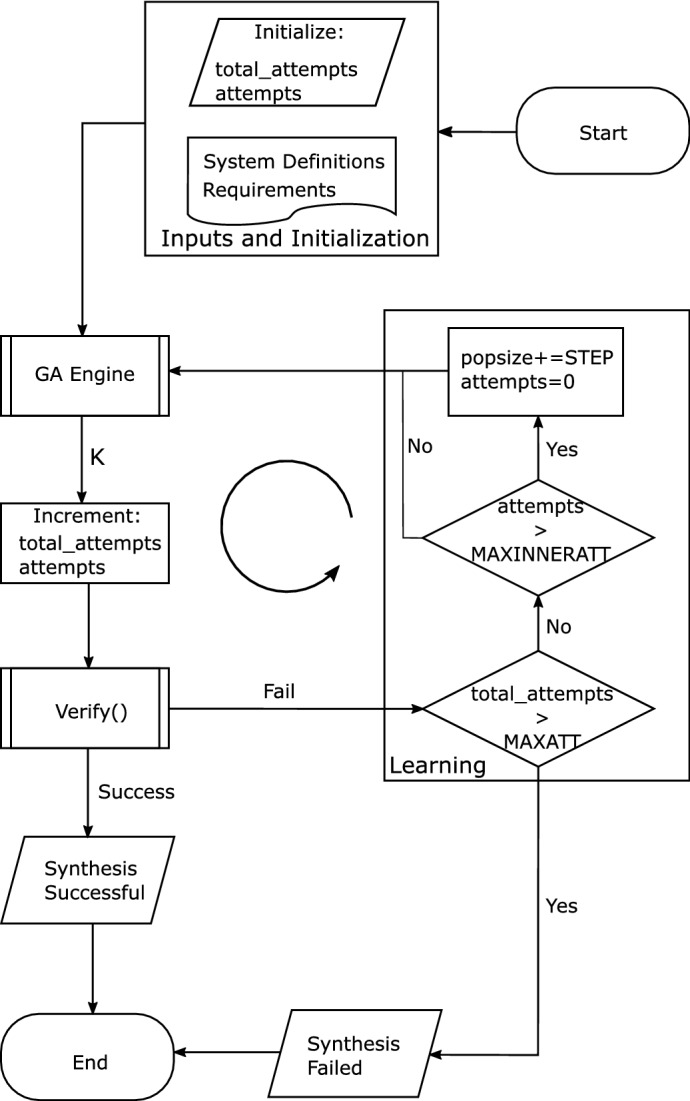
Proposed synthesis architecture.

In Fig. 4, we illustrate the proposed synthesis methodology, in a general form, where system definitions represent the overall system (state-space matrices and input). Moreover, the parameter requirements contains the requirements to be synthesized (settling time and/or overshoot), total_attempts is the total number of attempts to synthesize a controller, which is empirically defined by a user, attempts represents the number of attempts without changing any synthesis parameter (when a change occurs, a parameter is then reset and a new cycle is started, which also results in an increment to total_attempts), GA Engine is the block that performs the generation of a candidate controller through genetic algorithms, verify() is the verification engine (“Settling time and overshoot verification algorithms” section), which is basically the DSVerifier tool where we implemented our verification methodologies, MAXATT is the maximum number of attempts to synthesize a controller (as informed by a user), MAXINNERATT represents the maximum number of attempts without changing any GA parameter (MAXINNERATT is reset to zero, when a GA parameter is changed, while MAXATT continues to increment), popsize is the population size of the genetic algorithm, which was set to 100 chromosomes, as suggested by Roeva et al.^29^, and STEP is the step to increment the GA’s population, which, in the present work, was empirically obtained as 10. Besides, the proposed verification engine relies on what we are synthesizing (settling time and/or overshoot) and, as it is a generic framework, we could have also implemented different verification procedures and fed their results to the GA module.

In Fig. 4, we first define the system to be synthesized, which comprehends a plant, i.e., its state space matrices (${\mathbf {A}}$, ${\mathbf {B}}$, ${\mathbf {C}}$ and ${\mathbf {D}}$), a system’s input (we have used only the unit step), and the requirements a specified system should meet (settling time and/or overshoot). Next, we can step into *GA Engine*, where an optimization problem is implemented, based on a given system and considering the informed requirements as constraints (see details in “Generating a candidate controller K” section). Finally, as an output of *GA Engine*, a candidate controller *K*, which possibly makes a system satisfies the input requirements, is generated. Then, the verification process itself (according to the chosen property to synthesize) can now be performed, considering system definitions, the generated controller candidate, and the requirements to be verified (see “Verifying non-fragile performance specification requirements” section).

If the verification succeeds, then the associated synthesis procedure is successful and the entire process is terminated; otherwise, the total number of attempts (total_attempts) is evaluated to check if it exceeds the maximum number of attempts (MAXATT). If that is the case, the synthesis process fails and is terminated; otherwise, the number of attempts (attempts) is evaluated, without changing any GA parameter (e.g., population size), to check if it exceeds the maximum number defined for that (MAXINNERATT). If the latter is not valid, the population size of the GA is also incremented by a fixed step (STEP). Finally, GA Engine runs again, the entire process is repeated.

### Generating a candidate controller *K*

In our CEGIS-based synthesis methodology, we use genetic algorithms for generating a candidate controller *K*, in order to satisfy the desired requirements (overshoot and settling time). Our methodology uses two objective functions to solve three separated problems: synthesize for overshoot, settling time, or both. The objective functions are

9 $$\begin{aligned} \begin{array}{ccc} {\mathbf {f}}_\mathrm {1}(K)=K^{\top }K, \\ \end{array} \end{aligned}$$

10 $$\begin{aligned} \begin{array}{ccc} {\mathbf {f}}_\mathrm {2}(K)={\hat{k}}\\ \end{array}, \end{aligned}$$

where *K* is the controller matrix and ${\hat{k}}$ is given by Eq. (6).

We have chosen the objective function ${\mathbf {f}}_\mathrm {1}$ because of its simplicity and also to avoid a high noise sensitivity, which may happen as a result of significant gains (*K*). Therefore, minimizing ${\mathbf {f}}_\mathrm {1}$ implies minimizes the controller induced norm, which may help to avoid overshooting. Besides, ${\mathbf {f}}_\mathrm {2}$ is based on settling time calculation (${\hat{k}}$), which is easy to specify as an optimization problem. It is worth noticing that it is not mandatory to specify an objective function related to a specific constraint, although some intrinsic advantages may come from that. Indeed, we must only specify a function, while the associated solver (in the present case, a genetic algorithm) will try to find a solution, considering the input constraints. Other objective functions have indeed been tested, such as norms $H_2$ and $H_{\infty }$, but they have not provided good results due to the lack of relationship with input requirements and global outcome. Moreover, objective functions related to overshoot were not successful either.

#### Optimization problem to meet overshoot and settling time

In order to generate a controller *K*, using a genetic algorithm that satisfies both overshoot and settling time requirements, we formulate the following optimization problem:

11 $$\begin{aligned} \begin{array}{ccc} \min &{} ({\mathbf {f}}_\mathrm {1}(K),{\mathbf {f}}_\mathrm {2}(K)), \\ \\ \text { s.t. } &{} {{\hat{k}}} \le k_{\mathrm {sr}}, \\ &{} PO \le PO_{\mathrm {r}}, \\ &{} {\bar{\lambda }} \le 1. \\ \end{array} \end{aligned}$$

The constraints above are precisely what we look for, as they directly represent the desired requirements. The last constraint (${\bar{\lambda }} \le 1$) is crucial, given that an output system must be stable; indeed, without stability, performance analysis can not be executed. In the case a problem with different constraints is needed, i.e., other requirements, the given problem is specified in the same way, but an applicable rule that satisfies the underlying requirements must then be formulated. For instance, if a critically damped system is desired, the constraint $\zeta = 1$^2^ could be added.

In the present case, we want no minimize the multi-objective functions (${\mathbf {f}}_\mathrm {1}$ and ${\mathbf {f}}_\mathrm {2}$) constrained to ${\hat{k}} \le k_\mathrm {sr}$, $PO \le PO_{\mathrm {r}}$, and ${\bar{\lambda }} \le 1$. The first constraint guarantees that the system meets the settling time requirement, since $k_\mathrm {sr} \ge {\hat{k}}$ (cf. Algorithm 2). The second one, in turns, assures the required maximum percentage overshoot. Finally, the third one ensures that the controller *K* will keep a system stable, with ${\bar{\lambda }}$ being the largest absolute eigenvalue that is also smaller than 1, which provides stability.

It is worth to mention that the candidate controller generated by solving the problem (11) does not consider the FWL effects. However, the verification step of the proposed CEGIS algorithm considers the FWL effects by computing $K_{\mathrm {FWL}}:={\mathscr{F}}{\mathscr{W}}{\mathscr{L}}_{\mathrm {\langle I;F \rangle }}[K]$. Therefore, the verification step is based on Algorithms 1 and 2 for the system (3), and the synthesized controller *K* is considered safe only if the performance requirements hold for $K_{\mathrm {FWL}}$.

### Architecture of DSVerifier

The synthesis methodology presented above was implemented within the DSVerifier tool to experiment with the techniques developed in the present work in the context of several benchmarks. Moreover, such a development would enhance DSVerifier even further and make it a comprehensive tool for digital systems, but now regarding performance requirements and robustness aspects such as overflow, limit-cycle oscillation, and finite word-length^17^. The architecture illustrated in Fig. 4 was added to DSVerifier.

The synthesis part was implemented in C++, where all the necessary matrix operations were performed with the help of library Eigen^30^, while the GA module was implemented with the support of library Galgo^31^, also available for C++, which was used as an optimization tool for generating candidate controllers. The settling time and overshoot verification algorithms shown in “Verifying non-fragile performance specification requirements” section were already implemented within DSVerifier, due to a previous work^9^.

## Experimental evaluation

### Experimental objectives

In order to validate the methodology proposed in this work effectively, the related features were developed and implemented in C++ as part of DSVerifier. With their addition, we were able to submit our methodology to a set of benchmarks created exclusively for this work so that two original contributions are promptly identified. First, we provide a suitable test suite for future work. Second, we present results with tests targeting controllers synthesized for meeting performance requirements.

Apart from implicit contributions, our experiments have the following goals:

- EG1 **(Suitability)** Demonstrate the suitability of the proposed methodology regarding digital-controller synthesis;
- EG2 **(Performance)** Evaluate the effectiveness and the advantages of the proposed methodology when comparing it with other existing approaches.

### Experimental setup

The experiments presented here were carried out on four similar computers with slightly different configurations. That was done to speed up result generation, since the synthesis experiments executed here demand considerable time for their completion, as they consume many resources. If they were all executed on the same machine, the associated results would take very long to complete in parallel. The chosen machines have the following base configuration: a processor Intel (R) Core (TM) i7–4500 CPU @ 1.8 GHz, with 16 GB of RAM and running Ubuntu 64-bits. More informations to run the experiments can be found in the supplementary material.

### Description of the benchmarks

The benchmarks with the control systems used to evaluate the proposed approach are described below. In particular, they are available at Appendix with the information about state-space matrices ${\mathbf {A}}$, ${\mathbf {B}}$ and ${\mathbf {C}}$, required settling time $t_{\mathrm {sr}}$, and required percentage overshoot $PO_{\mathrm {r}}$. We have assumed that all benchmarks present a state-space matrix ${\mathbf {D}}=0$.

Those systems were chosen based on the possibility of providing different scenarios, in relation to the eigenvalues of ${\mathbf {A}}$. All experiments were conducted using the following configuration: ts = 0.5, p = 5, bounds_l = -0.5, bounds_u = 0.5, popsize = 100, and nbgen = 100. The first four parameters match what is used in practice, while the last two were empirically defined by performing extensive baseline tests. The performance of the proposed methodology, as implemented in DSVerifier, was then obtained with those same values, with the goal of successfully synthesizing controllers. In addition, there exist systems of 2nd (benchmarks 1, 5, 8, 12 and 15), 3rd (benchmarks 2, 9 and 13), 4th (benchmarks 3, 6, 7, 10 and 14), and 5th (benchmarks 4 and 11) order.

The chosen benchmarks present different eigenvalue configurations. Considering $\sigma _A$ the set of eigenvalues of matrix ${\mathbf {A}}$, there exist systems with $\sigma _A$ = {all $\lambda _{n}$ are the same, depending on a system’s order | $\lambda \in {\mathbb {R}}$}, $\sigma _A$ = {all $\lambda _{n}$ are different | $\lambda _{n} \in {\mathbb {R}}$ and/or $\lambda _{n} \in {\mathbb {C}}$}, and $\sigma _A$ = {$\forall \lambda _{n}$ | $\lambda \in {\mathbb {R}}$}, in our benchmark set. With this diversity, it is possible to cover scenarios that present different behaviors, such as overdamped, underdamped, and critically damped systems, in addition to second, third, fourth and fifth-order systems, with the goal of making a thorough analysis. Moreover, benchmark 15 is the model of a DC motor.

The chosen benchmarks were tested with 3 different FWL configurations (8, 16 and 32 bits), in the following scenarios: considering only settling time, only overshoot, and both properties simultaneously (settling time and overshoot). Consequently, that amounts to 45 tests per property scenario and 135 in total.

As a matter of avoiding corner cases and complex scenarios, which may present little practical use, we have empirically fixed the test time-out to 15 days. Consequently, if an individual experiment reaches a running time of 15 days, it is aborted.

### Experimental results

The Table 1 summarizes the experimental results for our benchmarks. The column **ID** identifies each benchmark, **Order** indicates the number of continuous variables, **R** represents the result of the associated synthesis (successful or failed), **T** is the number of attempts to perform a synthesis procedure, and, finally, $\langle 4,4 \rangle$, $\langle 8,8 \rangle$ and $\langle 16,16 \rangle$ represent the considered FWL format, where they represent a total of 8, 16, and 32 bits, respectively. When an experiment fails, Table 1 indicates MA or TO, which means the maximum number of attempts has been reached or there was time-out during a specific execution, respectively.

**Table 1:** We show the experimental results considering settling time and overshoot specifications.

**ID**	**Order**	Settling time						Overshoot						Settling time and overshoot					
		<4,4>		<8,8>		<16,16>		<4,4>		<8,8>		<16,16>		<4,4>		<8,8>		<16,16>	
		***R***	***T***	***R***	***T***	***R***	***T***	***R***	***T***	***R***	***T***	***R***	***T***	***R***	***T***	***R***	***T***	***R***	***T***
1	2	S	1	S	1	S	3	S	5	S	1	S	1	S	1	S	1	S	3
2	3	S	1	S	1	S	4	F	TO	S	1	S	1	S	1	S	1	S	4
3	4	S	1	S	2	S	2	S	1	S	1	S	1	S	1	S	2	S	2
4	5	S	1	S	1	S	1	S	1	S	1	S	1	S	1	S	1	S	1
5	2	S	1	S	1	S	7	S	19	S	1	S	1	S	1	S	1	S	7
6	4	S	1	S	5	S	41	S	5	S	1	S	1	F	TO	F	TO	F	TO
7	4	S	1	S	1	S	1	F	TO	F	TO	F	TO	F	TO	F	TO	F	TO
8	2	S	1	S	1	S	3	S	3	S	3	S	1	S	1	S	1	S	1
9	3	S	1	S	1	S	3	S	1	S	1	S	1	S	22	S	1	S	3
10	4	S	3	S	2	S	6	S	1	S	1	S	1	S	3	S	2	S	2
11	5	S	1	S	1	S	1	S	1	S	1	S	1	S	12	S	1	S	1
12	2	F	MA	F	MA	F	MA	F	MA	F	MA	F	MA	F	MA	F	MA	F	MA
13	3	F	MA	F	MA	F	MA	F	MA	F	MA	F	MA	F	MA	F	MA	F	MA
14	4	S	1	S	1	S	1	F	TO	F	TO	F	TO	F	TO	F	TO	F	TO
15	2	S	1	S	1	S	1	S	1	S	1	S	1	S	1	S	1	S	1

**ID** identifies each benchmark, **Order** indicates the number of continuous variables, **R** represents the result of the associated synthesis (successful or failed), **T** is the number of attempts to perform a synthesis procedure, **MA** means the maximum number of attempts has been reached, **TO** indicates that there was time-out during a specific execution, **S** means synthesis successful, **F** means synthesis failed.

On the one hand, the synthesis process performed by DSVerifier, which applies the methodology proposed in this work, presented, in total, 101 cases of successful synthesis, with 39 being experiments considering only settling time, where there were 13 success cases for each of the considered FWL configurations (8, 16, and 32 bits). Additionally, 32 experiments considering only overshoot were successful, where 10 employed 8 bits, 11 used 16 bits, and the rest were configured with 32 bits. Moreover, there were 10 successful cases for each FWL configuration (8, 16, and 32 bits), considering both settling time and overshoot, adding up 30 successful experiments to the total. On the other hand, there were 34 cases in which the proposed synthesis failed. Among those, 18 presented this result due to reaching the maximum number of pre-established attempts (MAXATT = 200), while 16 due to the synthesis execution time being exceeded (as mentioned in “Description of the benchmarks” section, the test time-out was fixed to 15 days). Thus, the maximum number of attempts was fixed to 200. This limit was empirically chosen during baseline experiments due to the obtained results, which usually presented worse figures when larger values were employed.

Indeed, there are two causes for those results, which we noticed during experiments and led to failures in synthesis processes: nature of closed-loop system’s eigenvalues and open-loop instability. Regarding long synthesizing times curbed by the imposed time-out value of 15 days, one apparent reason is the nature of a system’s eigenvalues, together with the number of bits used for the chosen FWL format. In summary, there exists no way to predict how long a synthesis process will take because each case presents its particularities.

**Figure 5:**
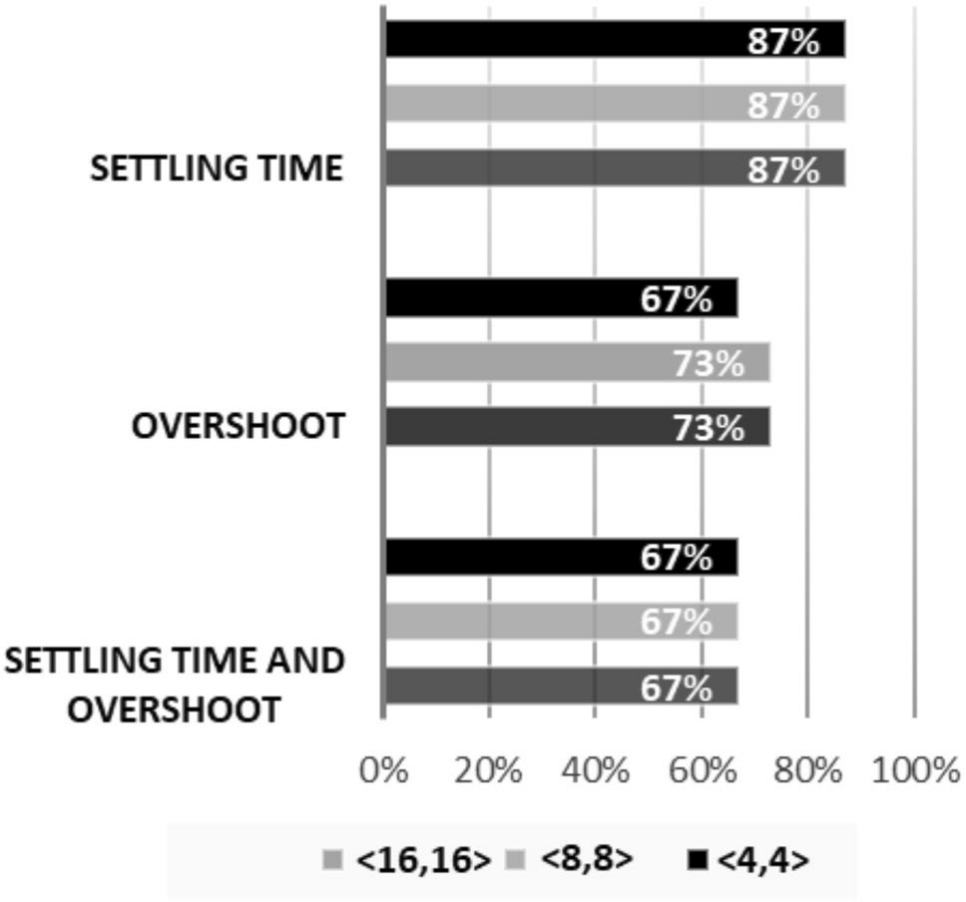
We present the percentage of success in experiments considering the settling time and overshoot specifications. We obtain the highest success rate (around 87%) when synthesizing digital controllers concerning settling time, while the experiments that tackle only overshoot varied from 67% to 73%, depending on the FWL format. Lastly, for the experiments considering both requirements, a success rate of 67% was obtained for all the analyzed FWL formats.

As illustrated in Fig. 5, experiments related to only settling time presented a success rate of 87%, when dealing with the 3 different FWL formats considered here ($\langle 4,4 \rangle$, $\langle 8,8 \rangle$, and $\langle 16,16 \rangle$). Regarding experiments tackling only overshoot, success rates of 73%, for formats $\langle 8,8 \rangle$ and $\langle 16,16 \rangle$, and 67%, for format $\langle 4,4 \rangle$ were achieved. Finally, concerning experiments considering both requirements, a success rate of 67% was obtained for all the analyzed FWL formats. Indeed, one may notice that combined requirements present a more complex scenario for the proposed synthesis scheme, with overshoot being more difficult than settling time. Moreover, the rates of successful attempts are mostly dictated by a specific requirement arrangement. Indeed, a system’s overshoot can be tightly controlled by tuning a controller’s gain^32^; however, it is still heavily influenced by a system’s eigenvalues and their relation with other parameters^33^, such as sampling time. Settling time figures, in turn, are mostly dominated by the largest eigenvalue^9^ in a system. Consequently, it becomes more challenging to control overshoot responses. The results generated by DSVerifier, i.e. the controller, were validated after each experiment, using Matlab, where we have simulated specific systems (i.e., state-space matrices and input), together with the synthesized controllers. Then, the obtained results were manually checked with the plot of the resulting step response to assure the desired settling time and/or overshoot values.

In experiments targeting only settling time, when applying FWL format $\langle 4,4 \rangle$, the synthesis was successfully carried out with one attempt, for benchmarks 1–9, 11, 14, and 15, and three attempts, for benchmark 10. Indeed, this is an interesting behavior for such experiments, given that, for instance, benchmark 11, which is a 5th-order system, needed only one attempt, as benchmark 1, which is a 2nd-order system. In summary, there is no clear relationship between FWL format, the nature of systems’ eigenvalues, and open-loop stability.

With FWL format $\langle 8,8 \rangle$, the successful cases carried out one, in case of benchmarks 1, 2, 4, 5, 7–9, 11, 14, and 15, two, in case of benchmarks 3 and 10, and five attempts, in case of benchmark 6. Again, there seems to be no clear relation between FWL format and the nature of a system’ eigenvalues, which makes some experiments need more attempts than others. However, there is a simple explanation for the behavior of benchmark 6, which will be clarified for the next format.

Experiments with FWL format $\langle 16,16 \rangle$ presented, in general, a more significant number of attempts than the other formats, which is indeed intuitively expected, where they needed one to forty-one attempts to successfully synthesize benchmarks 1–11, 14, and 15. Most of them needed more than one attempt to be successfully synthesized. A compelling case intrigued us: benchmark 6 needed 41 attempts, and the reason for that is not related to the nature of its eigenvalues (complex numbers) its open-loop instability. The combination of a more significant number of bits and eigenvalues profile created, for this specific benchmark, a state space system which took longer to be explored by *GA Engine*, as also happened with format $\langle 8,8 \rangle$. Indeed, the latter required an intermediate number of attempts, which clearly influences the chosen FWL format.

In order to provide a more transparent evaluation regarding synthesis attempts, the weighted average was used as a merit figure, which can be computed as

12 $$\begin{aligned} ATT_{AVG}=\frac{\sum _{n=0}^{N} G_{n}\times ATT_{n}}{\sum _{n=0}^{N} G_{n}}, \end{aligned}$$

where $ATT_{AVG}$ is the average number of attempts, $G_{n}$ is the group *n* of benchmarks with the same number of attempts, and $ATT_{n}$ is the number of attempts for this same group. Format $\langle 4,4 \rangle$ took 1.15, format $\langle 8,8 \rangle$ took 1.46, and, finally, format $\langle 16,16 \rangle$ took 5.69 attempts, in average. Consequently, with settling time, larger FWL formats require more attempts.

**Figure 6:**
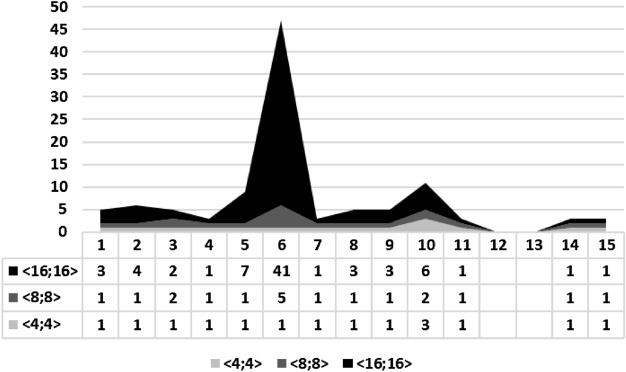
We illustrate the area chart for our settling time experiments under different FWL formats ($\langle 4, 4 \rangle$, $\langle 8, 8 \rangle$, and $\langle 16, 16 \rangle$) and the number of attempts to synthesis the digital controllers from 1 to 15. As we can observe, the FWL format directly influences the number of attempts to synthesize these digital controllers considering the settling time specification.

This same trend is also noticed by using an area chart to relate the number of attempts in each experiment and chosen FWL format. The ordinate axis shows the number of attempts for synthesizing a given controller that meets specific requirements. At the same time, the **ID** of the experiments is described on the abscissa axis, as illustrated in Fig. 6.

The gaps in Fig. 6 refer to the developed tool not being able to synthesize benchmarks successfully due to reaching the established maximum limit of attempts. In those cases, i.e., benchmarks 12 and 13, that happened due to poles that lead to system instability, in open-loop.

Considering only overshoot, experiments with format $\langle 4,4 \rangle$ took between one and nineteen attempts to successfully execute synthesis procedures regarding benchmarks 1, 3–6, 8–11, and 15. Moreover, the other formats ($\langle 8,8 \rangle$ and $\langle 16,16 \rangle$) took only one attempt to be successfully synthesized, with benchmarks 1–6, 8–11, and 15, mainly because most of them have real eigenvalues, which led to outputs without a large ripple.

Regarding overshoot experiments, $ATT_{AVG}$ figures of 3.8, 1.0, and 1.0 attempts were obtained for formats $\langle 4,4 \rangle$, $\langle 8,8 \rangle$, and $\langle 16,16 \rangle$, respectively. Although this reverse behavior may initially seem strange, it is indeed expected: coarser quantization strategies may impair algorithm convergence. As already mentioned, the overshoot is heavily influenced by a system’s eigenvalues and their relation with other parameters and, in the present case, a coarser representation led to situations where the exact coefficient values (their combination) were not found, for expected behavior, due to larger gaps between consecutive quantized numbers, which ultimately resulted in a higher number of attempts.

**Figure 7:**
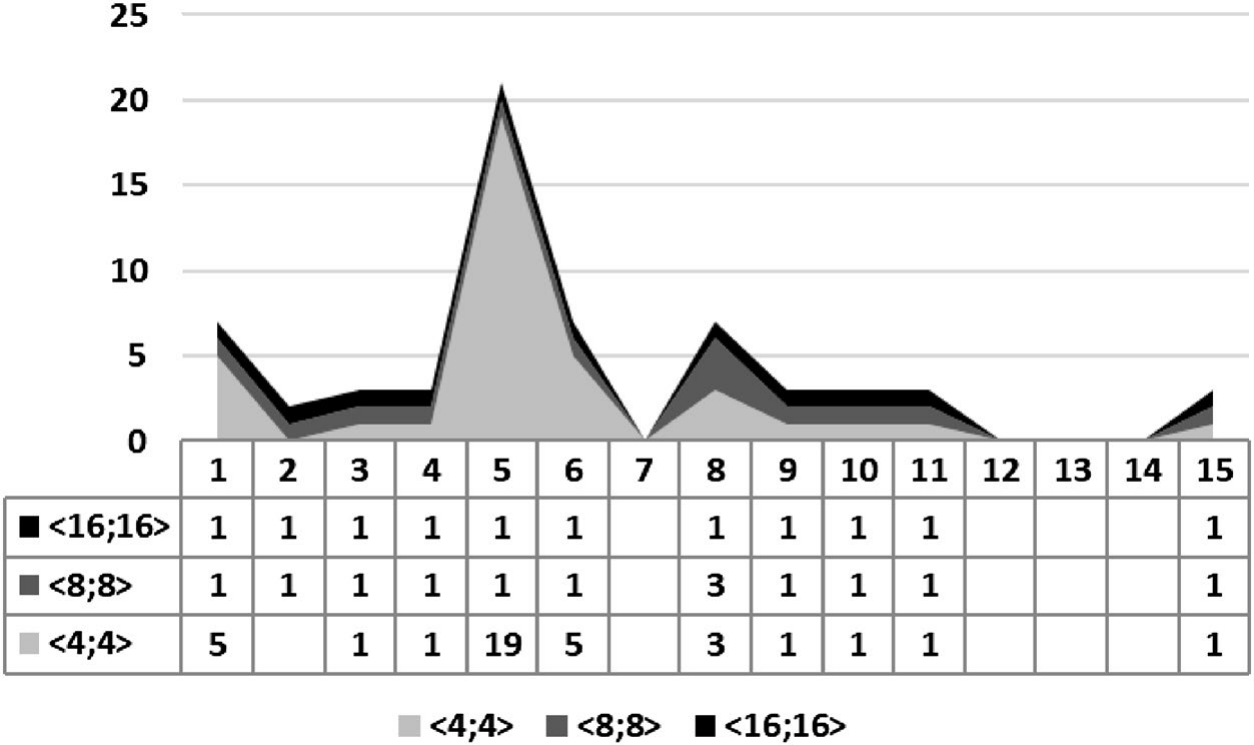
We illustrate the area chart for our overshoot experiments under different FWL formats ($\langle 4, 4 \rangle$, $\langle 8, 8 \rangle$, and $\langle 16, 16 \rangle$) and the number of attempts to synthesis the digital controllers from 1 to 15. As we can observe, the FWL format directly influences the number of attempts to synthesize these digital controllers considering the overshoot specification.

The area chart in Fig. 7 shows the opposite behavior noticed earlier. Indeed, benchmarks 1, 5, and 8, with format $\langle 4,4 \rangle$, presented a more significant number of attempts for successful synthesis procedures, while benchmark 8, with format $\langle 8,8 \rangle$, required as many attempts as those for format $\langle 4,4 \rangle$. One could also notice that benchmark 5 demanded many attempts (19) during synthesis when considering format $\langle 4,4 \rangle$), which is related to its complex eigenvalues. Unlike experiments considering settling time, benchmark 5 had a more significant number of attempts for a smaller amount of bits. We did not find any plausible explanation for that behavior during the experiments.

When experiments considering both requirements (settling time and overshoot) were performed, in FWL format $\langle 4,4 \rangle$, they took between one and twenty-two attempts, with benchmarks 1–5, 8–11, and 15. It is interesting to mention that benchmarks 9 to 11 have 3 eigenvalues in common ($-0.2$, $-0.3$ and $-0.7$), and the reason for more attempts to synthesize those lies in the combination of those eigenvalues, when dealt with by *GA Engine*.

When format $\langle 8,8 \rangle$ was used, one or two attempts were necessary to successfully synthesize benchmarks 1–5, 8–11, and 15. Specifically, only benchmarks 3 and 10, both of 4th order, needed more than one attempt to be successfully synthesized.

In format $\langle 16,16 \rangle$, experiments took from one to seven attempts, with benchmarks 1–5, 8–11, and 15. The majority of those experiments needed more than one attempt to be successfully synthesized.

In experiments with formats $\langle 4,4 \rangle$, $\langle 8,8 \rangle$, and $\langle 16,16 \rangle$, $ATT_{AVG}$ figures of 4.4, 1.2, and 2.5 attempts were obtained. Although there seems to be no trend, a simple analysis regarding attempts for single requirements reveals what happened: for format $\langle 4,4 \rangle$, overshoot led to a high number of average attempts, which also happened to format $\langle 16,16 \rangle$, but now due to settling time. Finally, format $\langle 8,8 \rangle$ presented an intermediate number of average attempts, which is true for single requirements.

**Figure 8:**
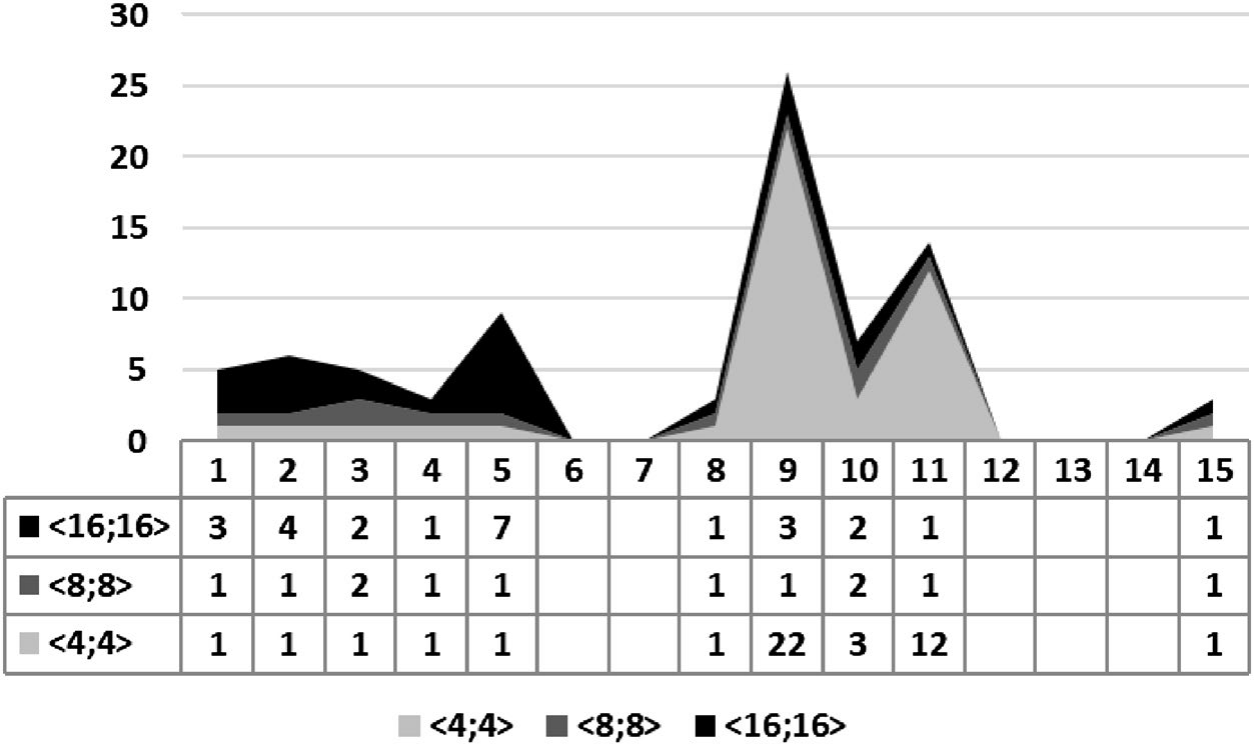
We illustrate the area chart for our experiments considering both settling time and overshoot under different FWL formats ($\langle 4, 4 \rangle$, $\langle 8, 8 \rangle$, and $\langle 16, 16 \rangle$) and the number of attempts to synthesis the digital controllers from 1 to 15. As we can observe, the FWL format directly influences the number of attempts to synthesize these digital controllers, considering settling time and overshoot as specification.

On the one hand, the area chart in Fig. 8 shows that benchmarks from 1 to 5 behave similarly to what can be noticed in Fig. 6, where more restricted FWL formats require fewer attempts. The reason behind this is that a coefficient combination for satisfying the overshoot requirement is quickly found, and, this way, settling time ends up being the dominant goal. On the other hand, benchmarks from 9 to 11 behave similarly to what is found in Fig. 7. Again, the smaller the number of bits in an FWL, the more attempts are needed to synthesize a controller, whose explanation lies on a dominant overshoot requirement. As a general consideration, an experiment behavior depends on the dominant requirement and possible combinations for achieving the desired figure, which, if known, might lead to a previous parameter tuning (e.g., *FWL format and settling time/overshoot value*). That indeed paves the way for a new study involving the creation of specification files. Nonetheless, one may notice that there are more unsuccessful procedures related to the synthesis of combined requirements.

Among groups of experiments organized by requirement configuration (settling time, overshoot, and both), we found out that overshoot ones, in average, took 1.93 attempts to synthesize a controller, followed by experiments considering both parameters, with 2.7, and settling time, with 2.77. Of course, the number of bits for an FWL format also impacts our tool in terms of attempts, as already shown.

One may notice that the synthesis tool failed to synthesize a controller that meets the requirements for benchmarks 12 and 13, whether considering only settling time, overshoot, or both. Such experiments failed due to reaching the established maximum-attempts limit, i.e., MAXATT = 200). A direct cause is the presence of unstable open-loop systems. In that case, the proposed methodology cannot find a controller that meets stability and performance requirements together. Indeed, *GA engine* (see Fig. 4) is unable to generate candidates that meet such requirements at the optimization problem level and still ensure stability.

Regarding cases where the proposed methodology with the chosen parameters and requirements was not able to synthesize a controller because the chosen time-out or the maximum attempts was reached, we have tried to change some parameters for them, such as the MAXATT and the time-out itself (in some cases, it was fixed to 30 days). Nonetheless, that was still not enough.

In addition, our tool failed in synthesizing benchmarks 2, 6, 7, and 14, for some combinations of format and requirements, due to exceeding the execution time limit. This fact can be related to the nature of systems’ eigenvalues, which are specifically complex eigenvalues. In that sense, other GAs with different strategies could also be employed, which is regarded as an extension to the present study.

In our experiment, we noticed that our methodology is more effective when synthesizing controllers for meeting only one requirement: settling time (39 successful and 6 failed) or overshoot (12 successful and 13 failed). Therefore, we still need to improve our methodology, regarding such a context, by using some effective multi-objective optimization tools.

### Threats to validity

We have shown a favorable assessment of our method over a set of digital control systems. Nonetheless, those benchmarks are limited within this paper’s scope, which means the proposed method’s performance should be further assessed on a more extensive set of real-world systems. Besides, we have a few parameters to be configured, such as the maximum number of attempts, which may lead to unsuccessful results, e.g., if the chosen maximum number of attempts is not sufficient for synthesizing a controller. The same happens to the parameters related to population size and the number of generations for the GA module, given that they can directly influence results. Nonetheless, in this paper, we indicate values for those parameters that we found suitable for the current experiments.

Moreover, we did not take into account the execution time needed to solve a specific problem. Instead, we have concentrated our effort on finding a correct controller able to make a system that meets a set of requirements. Consequently, our methodology can be time-consuming, depending on the system to be synthesized and the chosen parameters. As a consequence, one may argue that a consequent and necessary study regards parameter configuration based on target requirements and operation scenario, which may lead to an algorithm for generating *SpecsFile.ss*.

### Experiments with power converter models

We have chosen two power converter system examples: buck^34^ and boost^35^ converters. They demonstrate our formal synthesis methodology in physically motivating examples. In particular, those converters regulate the voltage and interconnect electrical systems. They are vital elements in the green economy due to their intensive application to low/zero carbon energy generation systems and consumers^36–38^.

#### Buck converter system

**Figure 9:**
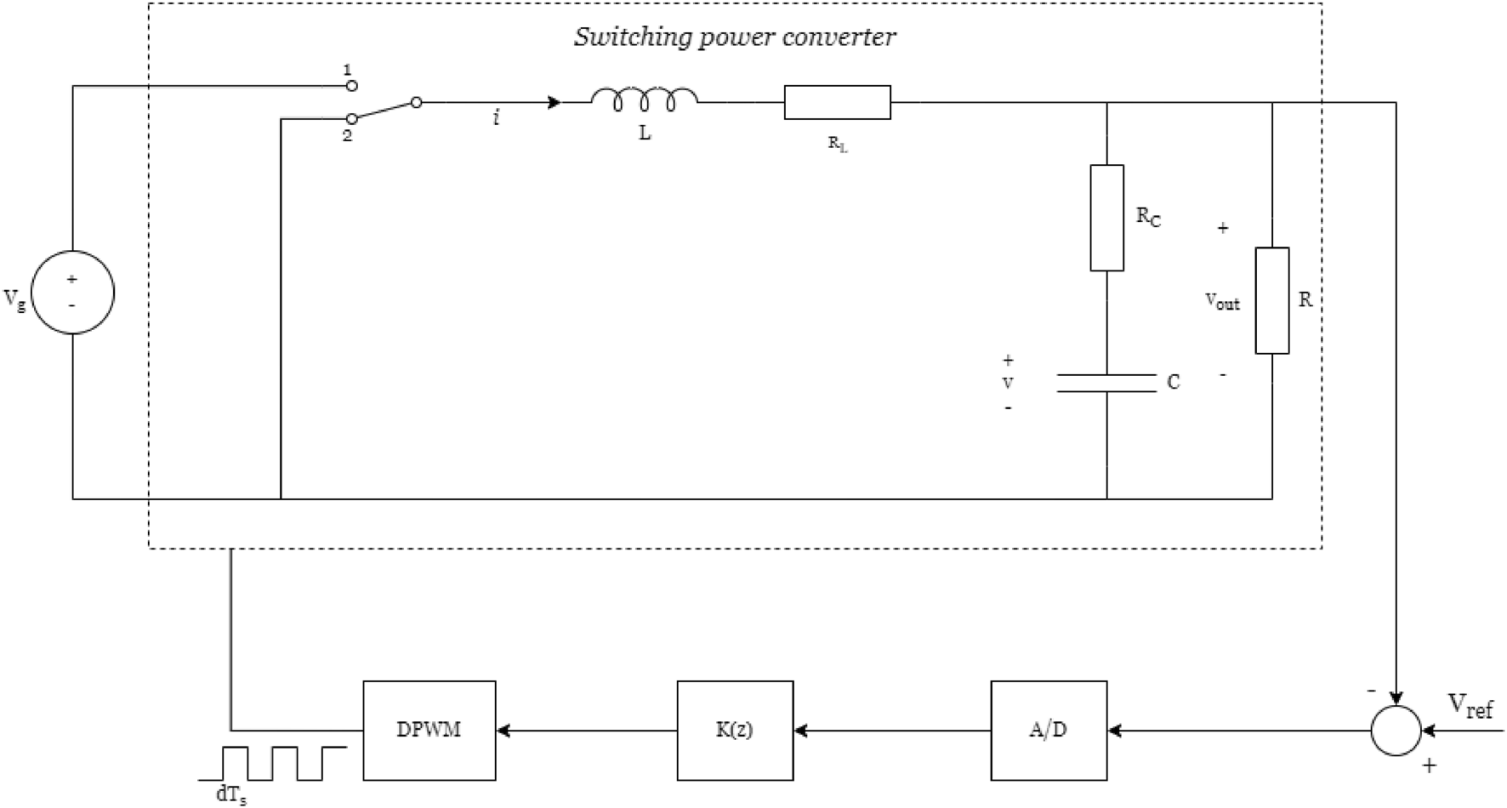
Buck Converter with Digital Control.

Buck converter (step-down converter)^34^ is a DC/DC converter that decreases the voltage (while increasing the current) from its input (power supply) to its output (load). Buck converters are used, for example, to reduce the voltage of laptop batteries (12-24V), providing the few volts necessary for modern processors to work such as Apple M2’s or Intel’s (x86)^39^. Figure 9 depicts the block diagram of a typical buck converter control loop.

To experiment with this system against our methodology, we present the state-space model for a Buck converter extracted from Iordanov & Halton^40^. The state-space model is described as follows.

13 $$\begin{aligned} \Omega _{buck}:{\left\{ \begin{array}{ll} x(k+1)=\begin{bmatrix} 1-\frac{T_s}{RC} &{} \frac{T_s}{C} \\ \frac{-T_s}{L} &{} 1-\frac{T_S R_C}{L} \end{bmatrix} x(k)+\begin{bmatrix} \frac{(T_s - t_d)V_g T_s}{LC} \\ \frac{V_g T_s}{L}-\frac{(T_s t_d)V_g T_s R_C}{L^2} \end{bmatrix}u(k),\\ y(k)=\begin{bmatrix} \frac{R}{R+R_C} &{} \frac{R_C R}{R_C + R} \end{bmatrix}x(k), \end{array}\right. } \end{aligned}$$

where *R*, *L*, *C*, $R_L$, $R_C$, $V_g$, $T_s$ and $t_d$ are, respectively, the load resistance, inductance, capacitance, line resistance, DC-link resistance, the power supply voltage, sampling time, and the delay time induced by the sampling process. In this paper, the values of those parameters are borrowed from Iordanov & Halton^40^ and are provided in Table 2 for convenience.

**Table 2:** Buck converter parameters with the respective nominal value.

Parameter	Nominal value
*R*	1.6 $\Omega$
*L*	500 nH
*C*	470 $\upmu$F
$R_L$	25 m$\Omega$
$R_C$	3 m$\Omega$
$V_g$	12 V
$T_s$	1 $\upmu$s
$t_d$	500*ns*

#### Boost converter system

A boost converter^35^ is used to “step-up” an input voltage to some higher level required by a load. This unique capability is achieved by storing energy in an inductor and releasing it to the load at a higher voltage. This capability highlights some of the more common pitfalls when using boost regulators. These include maximum achievable output current and voltage, short circuit behavior, and fundamental layout issues. We present a discrete state-space model for a Boost converter, which was directly extracted from Alkrunz et al.^41^. The state-space model is described as follows.

14 $$\begin{aligned} \Omega _{boost}:{\left\{ \begin{array}{ll} x(k+1)=\begin{bmatrix} 0.9968 &{} -0.0663 \\ 0.0955 &{} 0.9882 \end{bmatrix} x(k)+\begin{bmatrix} 6.9671 \\ -0.5687 \end{bmatrix}u(k),\\ y(k)=\begin{bmatrix} 0 &{} 1 \end{bmatrix}x(k) \end{array}\right. } \end{aligned}$$

All these values in the equation were taken from the work developed by Alkrunz et al.^41^.

#### Results

We conducted these experiments using our formal synthesis methodology implemented on the DSVerifier tool^17,42^ to synthesize a buck and a boost converter systems. In addition, we considered a required settling time and/or overshoot for such models (c.f. Eqs. 13, 14).

**Table 3:** Experiments for settling time only.

Name	FWL	Result	Controller	Attempts	ET (dd:hh:mm:ss)
Buck converter	$\langle 4,4 \rangle$	SUCCESFUL	[0.312500, 0.062500]	6	7:00:48:00
Buck converter	$\langle 8,8 \rangle$	SUCCESFUL	[0.4687500000, 0.0078125000]	1	1:03:35:00
Buck converter	$\langle 16,16 \rangle$	SUCCESFUL	[0.437271118178390983, 0.011184692383179000]	1	1:04:53:00
Boost converter	$\langle 4,4 \rangle$	SUCCESFUL	[0.250000, 0.250000]	1	0:02:46:00
Boost converter	$\langle 8,8 \rangle$	SUCCESFUL	[0.3085937500, 0.2265625000]	1	0:02:01:00
Boost converter	$\langle 16,16 \rangle$	SUCCESFUL	[0.302810668955235007, 0.155609130864474005]	1	0:02:21:00

It contains information about the converter name, FWL format, synthesis result, the synthesized controller, number of attempts to synthesize the controller, and total synthesis time.

The state-feedback controllers are synthesized from the buck and boost converters models (cf. “Buck converter system” and “Boost converter system” sections). Here, we consider three-step response requirements: only maximum admissible settling time, maximum admissible overshooting, and maximum settling time and overshoot. Moreover, we consider the FWL effects in 3 different formats, namely $\langle 4,4 \rangle$, $\langle 8,8 \rangle$, and $\langle 16,16 \rangle$.

Table 3 summarizes the synthesis results, considering only the settling time property. We can see that our approach was capable of synthesizing controllers for either Buck and Boost converter systems and every FWL format ($\langle 4,4 \rangle$, $\langle 8,8 \rangle$, and $\langle 16,16 \rangle$). However, as we can see, with the lowest number of bits in the FWL format, we took longer to synthesize, especially the Buck converter, due to the variable’s range and the number of constraints to be checked by our approach. So, as we increase the FWL format, we also increase the number of candidate solutions that our synthesizer stage can produce, and our verifier stage can check to meet the specification.

**Table 4:** Experiments for overshoot only.

Name	FWL	Result	Controller	Attempts	ET (dd:hh:mm:ss)
Buck converter	$\langle 4,4 \rangle$	SUCCESFUL	[0.250000, 0.062500]	1	1:16:12:00
Buck converter	$\langle 8,8 \rangle$	SUCCESFUL	[0.4726562500, 0.0078125000]	1	1:00:20:00
Buck converter	$\langle 16,16 \rangle$	SUCCESFUL	[0.449462890639727997 0.011352539062871999]	1	1:05:50:00
Boost converter	$\langle 4,4 \rangle$	SUCCESFUL	[0.312500, 0.250000]	1	0:03:19:00
Boost converter	$\langle 8,8 \rangle$	SUCCESFUL	[0.3125000000, 0.2890625000]	1	0:03:51:00
Boost converter	$\langle 16,16 \rangle$	SUCCESFUL	[0.314514160166555978, 0.312530517588366030]	1	0:03:37:00

It contains information about the converter name, FWL format, synthesis result, the synthesized controller, number of attempts to synthesize the controller, and total synthesis time.

Table 4 shows the results considering only the overshoot property. We can see that our methodology could synthesize digital controllers in all cases. However, for Buck converter experiments, we took longer to synthesize a controller to satisfy overshoot and FWL effects aspects due to the extensive state exploration produced by the underlying model and the property under verification.

**Table 5:** Experiments for both settling time and overshoot.

Name	FWL	Result	Controller	Attempts	ET (dd:hh:mm:ss)
Buck converter	$\langle 4,4 \rangle$	SUCCESFUL	[0.312500, 0.062500]	29	21:16:04:00
Buck converter	$\langle 8,8 \rangle$	SUCCESFUL	[0.4843750000, 0.0078125000]	1	1:01:05:00
Buck converter	$\langle 16,16 \rangle$	SUCCESFUL	[0.486404418961251028, 0.011489868164439001]	1	1:10:12:00
Boost converter	$\langle 4,4 \rangle$	SUCCESFUL	[0.250000, 0.187500]	1	0:02:04:00
Boost converter	$\langle 8,8 \rangle$	SUCCESFUL	[0.3125000000, 0.2812500000]	1	0:03:22:00
Boost converter	$\langle 16,16 \rangle$	SUCCESFUL	[0.304702758799047013, 0.188201904303042011]	1	0:02:47:00

It contains information about the converter name, FWL format, synthesis result, the synthesized controller, number of attempts to synthesize the controller, and total synthesis time.

It is worth mentioning that both buck and boost converters exhibit fast dynamics with poor damping. Thus, synthesizing state-feedback controllers to guarantee some settling time property, as reported in Table 3, is not a challenging task. Furthermore, although the damping is poor, meeting only overshoot requirements is also easily achieved by sacrificing the response speed since those converters are open-loop stable systems. However, meeting tight settling time and overshooting requirements simultaneously become challenging.

Table 5 shows the results considering both settling time and the overshoot properties. This experiment helped evaluate our proposed synthesizer because in the Buck converter, considering both properties and FWL effects, our tool tried to synthesize a digital controller. However, only after the 29th attempt could it synthesize a satisfiable controller. It happens because our synthesizer stage struggles to produce candidate solutions using the GA algorithm, considering the underlying state-space model that can meet the given specification. In this respect, the verifier stage must also check more challenging verification conditions, considering settling time and overshoot properties.

**Figure 10:**
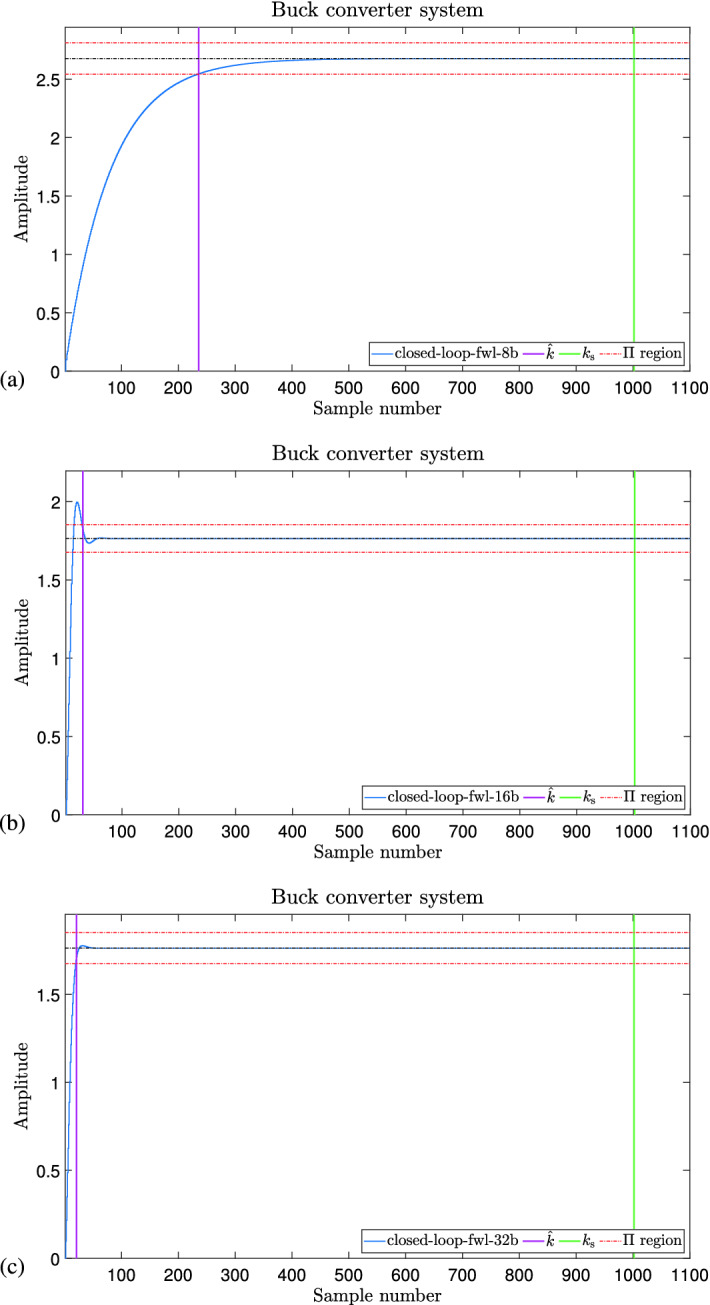
Response output of the buck converter with different FWL effect formats, showing that our controllers meet the requirements. (**a**) FWL $\langle 4,4 \rangle$ (**b**) FWL $\langle 8,8 \rangle$ (**c**) FWL $\langle 16,16 \rangle$.

Figure 10 shows the response output for the buck converter system illustrated in “Buck converter system” section. This response refers to the synthesized controller considering both settling time and overshoot specification and FWL effects of the form $\langle 4,4 \rangle$ in (a), $\langle 8,8 \rangle$ in (b), and $\langle 16,16 \rangle$ in (c). As we can see, DSVerifier, using the proposed methodology, could successfully synthesize a controller in all cases ($\langle 4,4 \rangle$, $\langle 8,8 \rangle$, and $\langle 16,16 \rangle$). In our experiment, we required a settling time of 1 ms and an overshoot of $45\%$, and all three synthesized controllers met the requirements under FWL effects. In the 3 cases, for the required settling time, the response is already in the settling time region, i.e., it meets the requirement. In addition, the overshoot in all 3 cases is under the maximum required in these experiments. If we consider Fig. 4, we have a main loop where the learning module (genetic algorithms) generates a candidate controller to test against our verification engine to see if it meets the requirements. In the case of this experiment, it required to loop 29 times for FWL format $\langle 4,4 \rangle$, and one time for the other two formats to find a controller that meets all the requirements, including the FWL effects, as we can see in Table 5 (Column Attempts).

**Figure 11:**
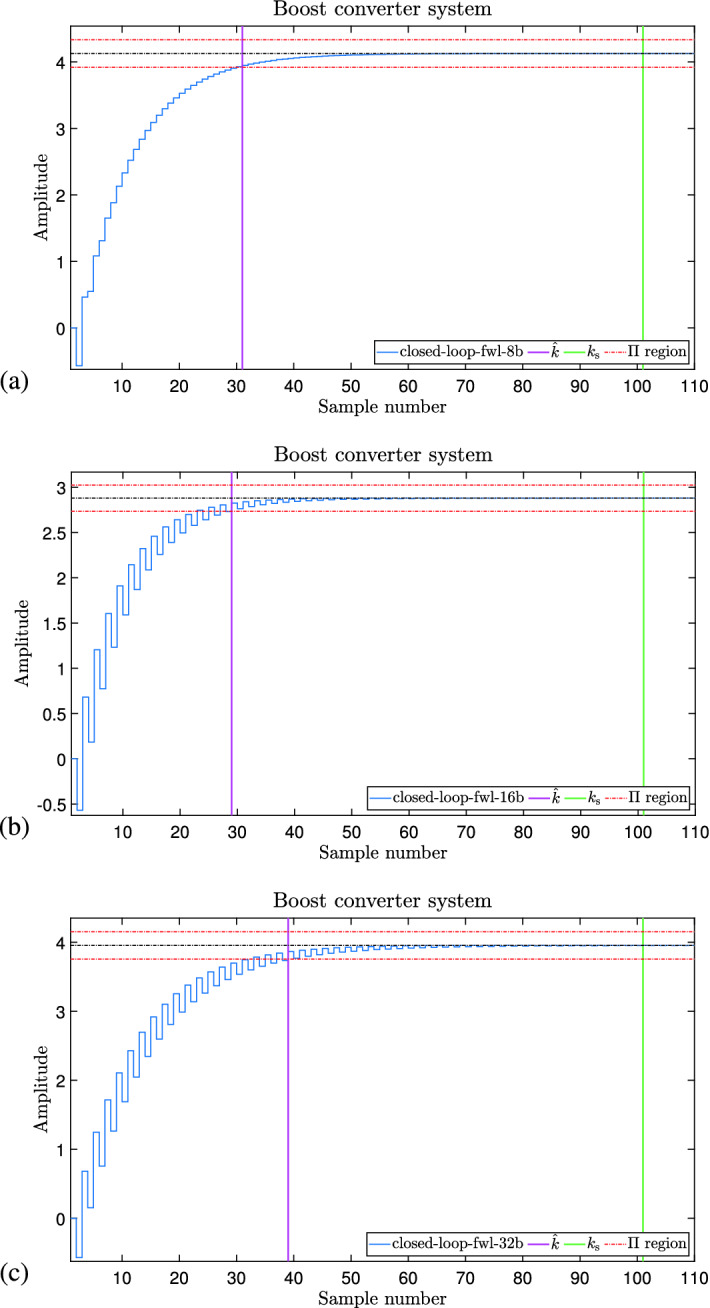
Response output of the boost converter with different FWL effect formats, showing that our controllers meet the requirements. (**a**) FWL $\langle 4,4 \rangle$ (**b**) FWL $\langle 8,8 \rangle$ (**c**) FWL $\langle 16,16 \rangle$.

Figure 11 shows the response of the Boost converter system in “Boost converter system” section, when the synthesized controller is applied. In all 3 cases—Fig. 11a–c, the required settling time ($k_{sr}=t_{sr}/T_s$) is already in the settling time region (as the response reached the settling time region, it did not leave it anymore), which means that the system meets this specific requirement. Adding to that, we also see that the overshoot in the response is under the required one, which leads to meeting this requirement as well (calculated overshoot less than $1\%$, much smaller than the required one—$45\%$). For all FWL formats evaluated in this work, our tool, according to Fig. 4, only needed one attempt to generate a controller that met all the requirements.

One vital observation we noticed in the results is related to the experiment with the Buck converter, considering both settling time and overshoot and the FWL effects in format $\langle 4,4 \rangle$. In that experiment, DSVerifier took total_attempts$=29$ to synthesize a controller that met the requirements, representing 21 days to finish the synthesis process. The reason why that happened is intriguing, given that for the other formats of FWL, the methodology did not have a problem synthesizing a controller (about one day and one attempt). It can be explained by the loss of performance due to the quantization. Indeed, it is easier to meet the step response requirements with more bits since it produces a more extensive set of candidate controllers. Thus, the synthesizer takes more time to produce a valid controller with the lowest number of bits in the FWL format because of consecutive failed attempts.

## Related work

The CEGIS synthesis technique can be regarded as the problem of computing correct-by-construction programs from a set of high-level specifications. In recent years, methods to solve this kind of problem have been used in different applications. For instance, Buchwald, Fried, and Hackin^43^ presented a fully automatic approach to create probably correct rule libraries, from formal specifications of an instruction set architecture and a compiler IR, using a hybrid approach that combines enumerative techniques with template-based CEGIS.

Abate et al.^23,44^ presented an approach to synthesizing safe digital feedback controllers for physical plants represented as linear time-invariant models. That approach uses the CEGIS technique, which has two phases. The first one synthesizes a static feedback controller that stabilizes a given system, but may not be safe for all initial conditions. Safety is then verified via BMC or abstract acceleration: if the verification step fails, a counterexample is provided to the synthesis engine and the process iterates until a safe controller is obtained.

Bloem et al.^45^ proposed an approach for the automatic synthesis of Byzantine-tolerant self-stabilizing systems in the form of distributed labeled transition systems. In this work, the synthesis method takes, as input, a description of the network of processes and a specification in linear-time temporal logic and a bound on the number of Byzantine processes in the same network. It encodes the existence of a solution into a problem in satisfiability modulo theories (SMT) and then tries to synthesize correct implementations for all processes if they do exist.

Lv, Zhang, and Zhang^46^ presented a robust stabilization algorithm based on a periodic observer for linear discrete-time periodic (LDP) systems. In their paper, the problem of state observer design is transformed into the solution to the corresponding matrix equation, and an iterative algorithm is given based on the conjugate-gradient (CG) algorithm. Initially, they consider the state observer design problem for linear discrete-time periodic systems without disturbances and give the expected algorithm. In that way, they consider the case where uncertain disturbances existed in a system’s parameters and provided an algorithm considering minimum norm and robustness.

Farahani et al.^47^ automatically synthesize reactive controllers for cyber-physical systems subject to signal temporal logic (STL) specifications. They explored three different methods for solving the required worst-case model predictive control (MPC) problem: a multi-parametric mixed-integer linear programming (MILP) solution, a Monte Carlo approach, and a dual optimization scheme. In addition, the main goal of this work is to obtain a controller that satisfies desired properties despite a potentially adversarial environment: the controller must therefore be robust to uncertain exogenous actions.

Abate et al.^14^ presented an approach to program synthesis that combined the strengths of a counterexample-guided inductive synthesizer with those of a theory solver. It is focused on one particular challenge for program synthesizers: the generation of programs that require non-trivial constants. Unfortunately, that work does not focus on the digital control system. Consequently, it does not consider FWL effects, showing the lack of research that provides the analysis that our work targets.

Eriksen et al.^48^ presented an on-line controller technique for a signalized intersection. The controller reads the current data from the radar sensors and uses it to learn a near-optimal controller at each control step. This strategy uses machine learning techniques to synthesize near-optimal traffic light controllers. The authors have shown that properly using more detailed information from radars can dramatically decrease the waiting times and queue lengths. This work presented to be very good for solving this kind of problem. However, it does not seem good enough for the application we are applying in the present work, given that it does not consider either performance parameters in the controller or FWL effects.

The presented related work tackles different system synthesis applications, while some are restricted to safe and stable controllers. Nonetheless, in control systems, performance requirements are of paramount importance in the design phase of controllers, and, sometimes, designers do need that a system response meets a particular settling time and/or overshoot. The main difference between those studies and the one presented here is that the latter tackles performance requirements for digital control systems together with FWL effects. Indeed, FWL effects impair the behavior of a system and can lead to critical mistakes, such as those caused by disregarding the chosen hardware for a given final implementation. In that sense, our methodology provides a correct-by-construction controller that already takes into account hardware issues (e.g., FWL effects). In addition, the fact that we use GAs, which are capable of good convergence^49^, means there is potential for finding a solution for different types of systems, given their optimization strategy.

## Conclusion

This work assumes the main goal: the development of a correct-by-design methodology suitable to the synthesis of digital controllers that meet performance requirements of control systems, i.e., settling time and overshoot, concerning the step response and already considering FWL effects.

To this end, the development of synthesis and verification methodologies was supported by some algorithms shown in this work, which helped on the formal verification process of performance requirements, considering the FWL effects. For the synthesis, such algorithms are used in the verification process, one of the CEGIS-methodology stages, as another module of this synthesis scheme. Specifically, a genetic algorithm was used to generate controller candidates.

As shown in “Experimental evaluation” section, the synthesis methodology implemented in the DSVerifier tool was able to synthesize digital controllers in a variety of control-system benchmarks specially created for the present work, which is also a contribution, i.e., a test database. The CEGIS technique was used as a synthesizing method in an iterative way, where, at each iteration, a candidate controller is generated with the aid of a genetic algorithm.

The experimental evaluations carried out in this work aim to show the application of the proposed synthesis methodology to digital controllers that should meet the requirements of settling time, overshoot, or both, simultaneously, already considering the FWL effects. The methodology developed in this work, implemented in C++, as a new functionality of DSVerifier, synthesized most of the benchmarks used here.

Further work should include the support to parametric uncertainties by using non-deterministic verification. In addition, the synthesis engine may benefit from using techniques ranging from machine learning to more robust formulations for generating candidates in the synthesis scheme. Finally, it is also possible to extend our methodology to MIMO systems.

## Supplementary Information

Supplementary Information.
