# Supplementary material for: Formal synthesis of non-fragile state-feedback digital controllers considering performance requirements for step response
Source: Sci Rep. 2022 Sep 14;12:15429. doi: 10.1038/s41598-022-19284-4 (PMC9474889; doi:10.1038/s41598-022-19284-4)
Supplement: Supplementary file 1 — Supplementary Information. [file 41598_2022_19284_MOESM1_ESM.pdf]

Supplementary information file

Thiago Cavalcante[1], Iury Bessa[1], Lucas C. Cordeiro[2], and Eddie de Lima Filho[1,3]

[1]Federal University of Amazonas, Brazil
[2]University of Manchester, United Kingdom
[3]TPV Technology, Brazil

August 4, 2022

1 Data availability

The tools and benchmarks related to the manuscript entitled ”Formal Synthesis of Non-fragile State-Feedback Digital Controllers Considering Performance Requirements for Step Response” are available in the Zenodo repository, `https://doi.org/10.5281/zenodo.5943514`. In particular, the user may run the scripts `bench_experiments.sh`, `bench_experiments2.sh`, `bench_experiments3.sh`, `bench_experiments4.sh`, and `bench_experiments5.sh` to reproduce the results reported in the paper.

2 Benchmarks

The benchmarks used in the experimental evaluation reported in Section 5 are presented in the Table 1.

Table 1: Description of the benchmarks used in our experiments.

**ID**	A	B	C	t_{sr} **(s)**	PO_{r}
1	$\begin{bmatrix}-0.5 & 1.0\\ 0.0 & -0.5\end{bmatrix}$	$\begin{bmatrix}0.0\\ 2.5\end{bmatrix}$	$\begin{bmatrix}0.0 & 2.6\end{bmatrix}$	2.5	5
2	$\begin{bmatrix}-0.5 & 1.0 & 0.0\\ 0.0 & -0.5 & 1.0\\ 0.0 & 0.0 & -0.5\end{bmatrix}$	$\begin{bmatrix}-0.4\\ 2.5\\ -0.8\end{bmatrix}$	$\begin{bmatrix}0.0 & 2.6 & 0.0\end{bmatrix}$	3.5	5
3	$\begin{bmatrix}-0.5 & 1.0 & 0.0 & 0.0\\ 0.0 & -0.5 & 1.0 & 0.0\\ 0.0 & 0.0 & -0.5 & 1.0\\ 0.0 & 0.0 & 0.0 & -0.5\end{bmatrix}$	$\begin{bmatrix}1.0\\ 2.5\\ 1.0\\ 1.0\end{bmatrix}$	$\begin{bmatrix}0.0 & 2.6 & 1.2 & 0.0\end{bmatrix}$	4.5	30
4	$\begin{bmatrix}-0.5 & 1.0 & 0.0 & 0.0 & 0.0\\ 0.0 & -0.5 & 1.0 & 0.0 & 0.0\\ 0.0 & 0.0 & -0.5 & 1.0 & 0.0\\ 0.0 & 0.0 & 0.0 & -0.5 & 1.0\\ 0.0 & 0.0 & 0.0 & 0.0 & -0.5\end{bmatrix}$	$\begin{bmatrix}-0.4\\ -0.6\\ 5.5\\ 1.0\\ -0.3\end{bmatrix}$	$\begin{bmatrix}0.0 & 2.6 & 0.5 & 1.2 & 0.0\end{bmatrix}$	5.5	20
5	$\begin{bmatrix}-0.5 & 0.4\\ -0.4 & -0.5\end{bmatrix}$	$\begin{bmatrix}0.0\\ 2.5\end{bmatrix}$	$\begin{bmatrix}0.0 & 2.6\end{bmatrix}$	3.0	5
6	$\begin{bmatrix}-0.5 & 0.4 & 1.0 & 0.0\\ -0.4 & -0.5 & 0.0 & 1.0\\ 0.0 & 0.0 & -0.5 & 0.4\\ 0.0 & 0.0 & -0.4 & -0.5\end{bmatrix}$	$\begin{bmatrix}0.0\\ 0.0\\ 2.5\\ 1.6\end{bmatrix}$	$\begin{bmatrix}0.0 & 2.6 & 0.0 & 2.0\end{bmatrix}$	5.0	30
7	$\begin{bmatrix}-0.5 & 0.4 & 0.0 & 0.0\\ -0.4 & -0.5 & 0.0 & 0.0\\ 0.0 & 0.0 & -0.8 & 0.4\\ 0.0 & 0.0 & -0.4 & -0.8\end{bmatrix}$	$\begin{bmatrix}-0.4\\ -0.6\\ 2.5\\ 1.6\end{bmatrix}$	$\begin{bmatrix}0.0 & 2.6 & 0.0 & 2.0\end{bmatrix}$	10.0	4
8	$\begin{bmatrix}-0.2 & 0.0\\ 0.0 & -0.3\end{bmatrix}$	$\begin{bmatrix}-0.6\\ 2.5\end{bmatrix}$	$\begin{bmatrix}0.0 & 2.6\end{bmatrix}$	1.5	8
9	$\begin{bmatrix}-0.2 & 0.0 & 0.0\\ 0.0 & -0.3 & 0.0\\ 0.0 & 0.0 & -0.7\end{bmatrix}$	$\begin{bmatrix}-0.8\\ -0.7\\ -0.5\end{bmatrix}$	$\begin{bmatrix}0.0 & 2.6 & 0.0\end{bmatrix}$	2.0	8
10	$\begin{bmatrix}-0.2 & 0.0 & 0.0 & 0.0\\ 0.0 & -0.3 & 0.0 & 0.0\\ 0.0 & 0.0 & -0.7 & 0.0\\ 0.0 & 0.0 & 0.0 & -0.9\end{bmatrix}$	$\begin{bmatrix}-0.4\\ 2.5\\ 1.0\\ -0.7\end{bmatrix}$	$\begin{bmatrix}0.0 & 2.6 & 1.2 & 0.0\end{bmatrix}$	5.0	30
11	$\begin{bmatrix}-0.2 & 0.0 & 0.0 & 0.0 & 0.0\\ 0.0 & -0.3 & 0.0 & 0.0 & 0.0\\ 0.0 & 0.0 & -0.7 & 0.0 & 0.0\\ 0.0 & 0.0 & 0.0 & -0.9 & 0.0\\ 0.0 & 0.0 & 0.0 & 0.0 & -0.5\end{bmatrix}$	$\begin{bmatrix}-0.5\\ -0.2\\ 2.5\\ 2.0\\ -0.8\end{bmatrix}$	$\begin{bmatrix}0.0 & 2.6 & 0.5 & 1.2 & 0.0\end{bmatrix}$	10.0	18
12	$\begin{bmatrix}4.5 & 1.0\\ 0.0 & 4.5\end{bmatrix}$	$\begin{bmatrix}0.0\\ 2.5\end{bmatrix}$	$\begin{bmatrix}0.0 & 2.6\end{bmatrix}$	8.0	10
13	$\begin{bmatrix}1.5 & 1.0 & 0.0\\ 0.0 & 1.5 & 1.0\\ 0.0 & 0.0 & 1.5\end{bmatrix}$	$\begin{bmatrix}-0.4\\ 2.5\\ -0.8\end{bmatrix}$	$\begin{bmatrix}0.0 & 2.6 & 0.0\end{bmatrix}$	10.0	9
14	$\begin{bmatrix}-0.5 & 0.4 & 0.0 & 0.0\\ -0.4 & -0.5 & 0.0 & 0.0\\ 0.0 & 0.0 & -0.8 & 0.4\\ 0.0 & 0.0 & -0.4 & -0.8\end{bmatrix}$	$\begin{bmatrix}-0.4\\ -0.6\\ -0.4\\ -0.6\end{bmatrix}$	$\begin{bmatrix}0.0 & 2.6 & 0.0 & 2.0\end{bmatrix}$	10.0	2
15	$\begin{bmatrix}-0.6386 & 0.4151\\ -0.0098 & 0.8266\end{bmatrix}$	$\begin{bmatrix}0.2442\\ 1.0745\end{bmatrix}$	$\begin{bmatrix}0.1807 & 0.2076\end{bmatrix}$	6.5	5
